# Engineered nanomaterials enhance drug delivery strategies for the treatment of osteosarcoma

**DOI:** 10.3389/fphar.2023.1269224

**Published:** 2023-08-21

**Authors:** Haorui Zhang, Ping Luo, Xiaojun Huang

**Affiliations:** ^1^ Department of Spine, Trauma Surgery, The First People’s Hospital of Guangyuan, Guangyuan, China; ^2^ Science and Technology Education Section, The First People’s Hospital of Guangyuan, Guangyuan, China

**Keywords:** osteosarcoma, nanomaterials, drug delivery, chemotherapy, adverse reactions

## Abstract

Osteosarcoma (OS) is the most common malignant bone tumor in adolescents, and the clinical treatment of OS mainly includes surgery, radiotherapy, and chemotherapy. However, the side effects of chemotherapy drugs are an issue that clinicians cannot ignore. Nanomedicine and drug delivery technologies play an important role in modern medicine. The development of nanomedicine has ushered in a new turning point in tumor treatment. With the emergence and development of nanoparticles, nanoparticle energy surfaces can be designed with different targeting effects. Not only that, nanoparticles have unique advantages in drug delivery. Nanoparticle delivery drugs can not only reduce the toxic side effects of chemotherapy drugs, but due to the enhanced permeability retention (EPR) properties of tumor cells, nanoparticles can survive longer in the tumor microenvironment and continuously release carriers to tumor cells. Preclinical studies have confirmed that nanoparticles can effectively delay tumor growth and improve the survival rate of OS patients. In this manuscript, we present the role of nanoparticles with different functions in the treatment of OS and look forward to the future treatment of improved nanoparticles in OS.

## 1 Introduction

Osteosarcoma is the most common type of bone tumor in children and adolescents, accounting for about 60% of primary malignant bone tumors (Jemal et al., 2005). In children and adolescents, OS occurs in 2–3 per million people per year (Li et al., 2023). OS originates from osteoblasts responsible for bone growth, and osteoblasts damage important areas of bone, causing the bone to not repair itself (Shao et al., 2022). OS grows in a complex environment consisting of osteoblasts, osteoclasts, blood vessels, immune cells, and extracellular matrix (Alfranca et al., 2015). Imbalance in the balance between tumor parenchymal cells and non-tumor cell stromal cells is the basis for OS development and metastasis (Corre et al., 2020). Osteoclast-mediated osteolysis is the main cause of reduced bone fragility (Avnet et al., 2008). OS not only causes defects and damage to bone, affecting the functional integrity of bone but also brings serious accompanying symptoms to patients, manifested as unbearable bone pain, hypercalcemia, and pathological fractures (von Schacky et al., 2022). OS has a very high degree of malignancy, is prone to metastasis in the early stage, has strong drug resistance, and has a very poor prognosis. The lung is the most common site of metastasis in OS, up to 74% (Zhang et al., 2019). OS is the second leading cause of death among adolescent tumor-related diseases (Siclari and Qin, 2010). Over the past four decades, the incidence of bone tumors has stabilized, but the mortality rate of bone tumors in patients with bone tumors has gradually increased due to their highly aggressive and metastatic nature (Zhu et al., 2022).

In recent years, the treatment of OS has been continuously explored and studied. However, treatment of OS is still quite limited (Sergi, 2021). Current conventional treatments for malignancy are chemotherapy, radiation therapy, and surgical resection (Wu et al., 2022a). Surgery can only remove the local tumor, but cannot cure the metastasis (Zhu et al., 2022). Chemotherapy is one of the most commonly used cancer treatments. However, due to drug resistance and tumor heterogeneity, likely, chemotherapy alone will not eliminate the tumor (Zhang et al., 2020). The commonly used clinical approach to malignant OS is neoadjuvant chemotherapy combined with surgery (Somaiah et al., 2022). Although this treatment modality has made good progress, survival for malignant OS has been significantly improved (Eaton et al., 2021). However, there are still some thorny problems, such as drug resistance, large side effects, and poor improvement in survival rates of metastatic patients. The treatment of malignant bone tumors is still a difficult problem to solve clinically. The key factors reducing the therapeutic effect of OS: 1) the resistance of OS cells to chemoradiotherapy; 2) Reducing the adverse reactions of chemotherapy drugs (Zhu et al., 2022).

With the development of nanomedicine, nanomaterials offer new opportunities to improve drug delivery and reduce their adverse effects (Xia et al., 2022a). Nanoexamples are widely used in the immunotherapy of tumors using their unique size, charge properties, and targeting properties (Wu et al., 2022a). The enhanced permeability retention (EPR) properties of tumor cells allow nanoparticles to remain in the tumor microenvironment for a long time (Morales-Orue et al., 2019). Nanoparticles encapsulate chemotherapy drugs, immune adjuvants, etc., which can achieve precise release of drugs and avoid drug decomposition and inactivity in blood vessels (Nguyen et al., 2020). Through reasonable design, the pharmacokinetics of nanoparticles can be improved, and their circulation time in the body can be enhanced to improve the therapeutic effect of OS (Yuan et al., 2023). The manuscript focuses on the application of nanoparticles with different functions in OS, and analyzes the prospect of nanoparticles in the treatment of OS.

## 2 pH-responsive nanoparticles

Surface charge plays an important role in signal transmission between nanoparticles and cells (Hu et al., 2020). The surface of tumor cells often exhibits negative electricity, which makes the positively charged nanoparticles have a strong attraction effect with it. However, the tumor microenvironment is often weakly acidic, which makes positively charged nanoparticles often less likely to come into direct contact with tumor cells. Acid-unstable acetal ligates exhibit ideal stability at physiological pH and exhibit instability under acidic conditions (Feng et al., 2020). Acetal linking enables nanoparticles to achieve pH transitions and deliver carriers to tumor cells. Cinnamaldehyde (CA) has been shown to have antibacterial, antioxidant, anti-inflammatory, hypoglycemic, and antitumor effects (Zong et al., 2022). CA has been reported to regulate the Wnt/β-catenin and phosphatidylinositol-3-kinase (PI3K)/AKT signaling pathways to inhibit the proliferation of OS cells and induce their apoptosis (Huang et al., 2020). However, the disadvantages of high toxicity and low solubility of CA limit its clinical application. Deng et al. (2023) made an amphiphilic CA prodrug from polyethylene glycol (mPEG), ethanol, and CA. Amphiphilic polymers can self-assemble into nano micelles in an aqueous solution with a diameter of 227 ± 16 nm and a zeta potential neutral at pH 7.4. Due to the protonation of ethanol, the surface charge of the nano micelles increases dramatically with the decrease in pH, and the zeta potential of the nano micelles becomes +6.25 mV at pH 7.9. This charge conversion facilitates the targeting of OS by nano micelles. *In vitro*, experiments have shown that nano micelles can release about 96% of CA in 5 h. Not only that, but nano micelles can also enhance the release of reactive oxygen species (ROS), promote apoptosis of OS cells and achieve powerful anti-tumor effects.

**FIGURE 1:**
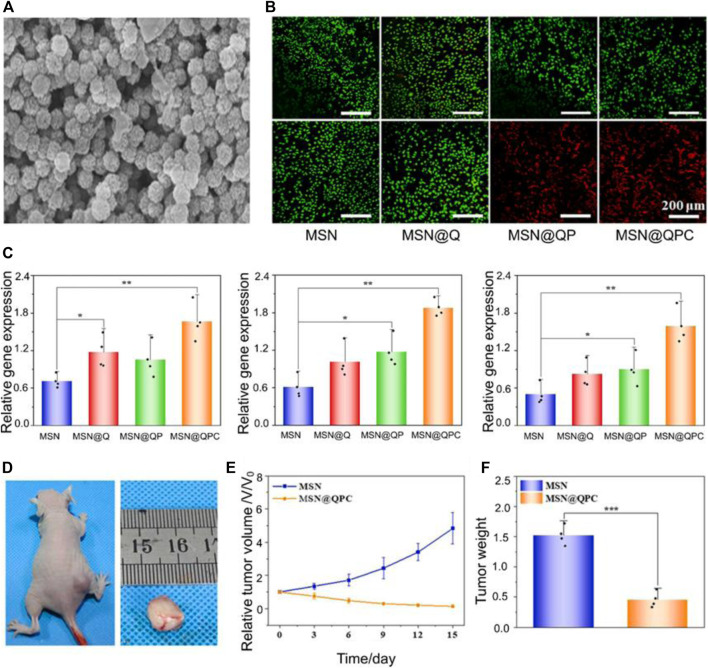
**(A)** Transmission electron microscopy image of MSN@QPC. **(B)** Cell Hoss staining: Causes more cell death (red: dead cells, green: live cells) in the case of MSN@QPC of light (bottom) than no light (top). **(C)** MSN@QPC Promotes extracellular matrix mineralization-related gene expression (CN, OPN, and RUNX2). **(D)** Tumor volume on day 15 of MSN@QPC treatment. **(E)** Relative tumor volume change. **(F)** Tumor weight on day 15. Reproduced with permission from (Shi et al., 2022).

Mesoporous silica nanoparticles (MSNs) play an integral role in nanomedicine due to their excellent high drug delivery efficiency, controlled drug release and biocompatibility (Tarn et al., 2013). Polydopamine (PDA) surfaces are rich in bioactive groups, and pH response can be achieved by improving their surface groups. Photothermal therapy is an emerging way of tumor treatment, the main principle is to irradiate the photothermal agent at a specific wavelength, so that the photothermal agent heats up and kills tumor cells (Zhang et al., 2013). Shi et al. (2022) designed a nanoparticle with pH responsiveness and photothermal performance (MSN@QPC) using MSN, PDA, and quercetin (Qr). At the same time, collagen is added, so that the nanoparticles have a unique role in inducing extracellular matrix deposition and enhancing osteogenic activity. MSN@QPC can inhibit tumor growth and improve the efficacy of PTT. The diameter of the MSN@QPC is 80 nm and has a high specific surface area (Figure 1A). The drug load rate of MSN@QPC was 31.73%. Due to the addition of PDA, the release of Qr is delayed MSN@QPC, and *in vitro* tests, 55% of Qr can be released in 72 h MSN@QPC, thus achieving a long-term release effect. The released Qr can cause an increase in the temperature in the OS cells without causing an increase in the temperature of the surrounding tissues under ultraviolet light irradiation of 4 nm. In the case of light, MSN@QPC causes tumor cell death without causing normal cell death (Figure 1B). Not only that, but MSN@QPC also promotes extracellular matrix mineralization (Figure 1C). MSN@QPC *in vivo* has been shown to enhance the killing effect of tumors (Figures D–F).

Yang et al. (2018) used ZSM-5 zeolite loaded with doxorubicin to make ZSM-5/CS/DOX core-shell nanodisks with chitosan as the shell. The Si element released in ZSM-5 zeolite contributes to osteoblast differentiation by antagonizing NF-κB activation (Zhou et al., 2016). The ZSM-5/CS/DOX core-shell nanodisk has a diameter of 100 nm, a pore size of 3.75 nm, and a drug loading rate of 97.7%. Due to the positive charge on the surface of chitosan, ZSM-5/CS/DOX core-shell nanodisks are pH responsive. At pH 6, ZSM-5/CS/DOX core-shell nanodisks can release 58.7% of DOX. DOX enters the tumor nucleus and interferes with the DNA proliferation of osteosarcoma to achieve anti-tumor effects (Maiuri et al., 2007). ZSM-5/CS/DOX core-shell nanodisks have a stronger anti-tumor effect than pH 7.4 at pH 5. This feature is more conducive to the role of nanoparticles in the acidic tumor microenvironment. Not only that, compared with intravenous DOX, ZSM-5/CS/DOX core-shell nanodiscs release DOX in 24 h in the body, which is high around the tumor and low in the heart. This phenomenon indicates that ZSM-5/CS/DOX core-shell nanodisks have a higher targeting effect and can reduce the cardiotoxicity of DOX.

## 3 ROS-responsive nanoparticles

**FIGURE 2:**
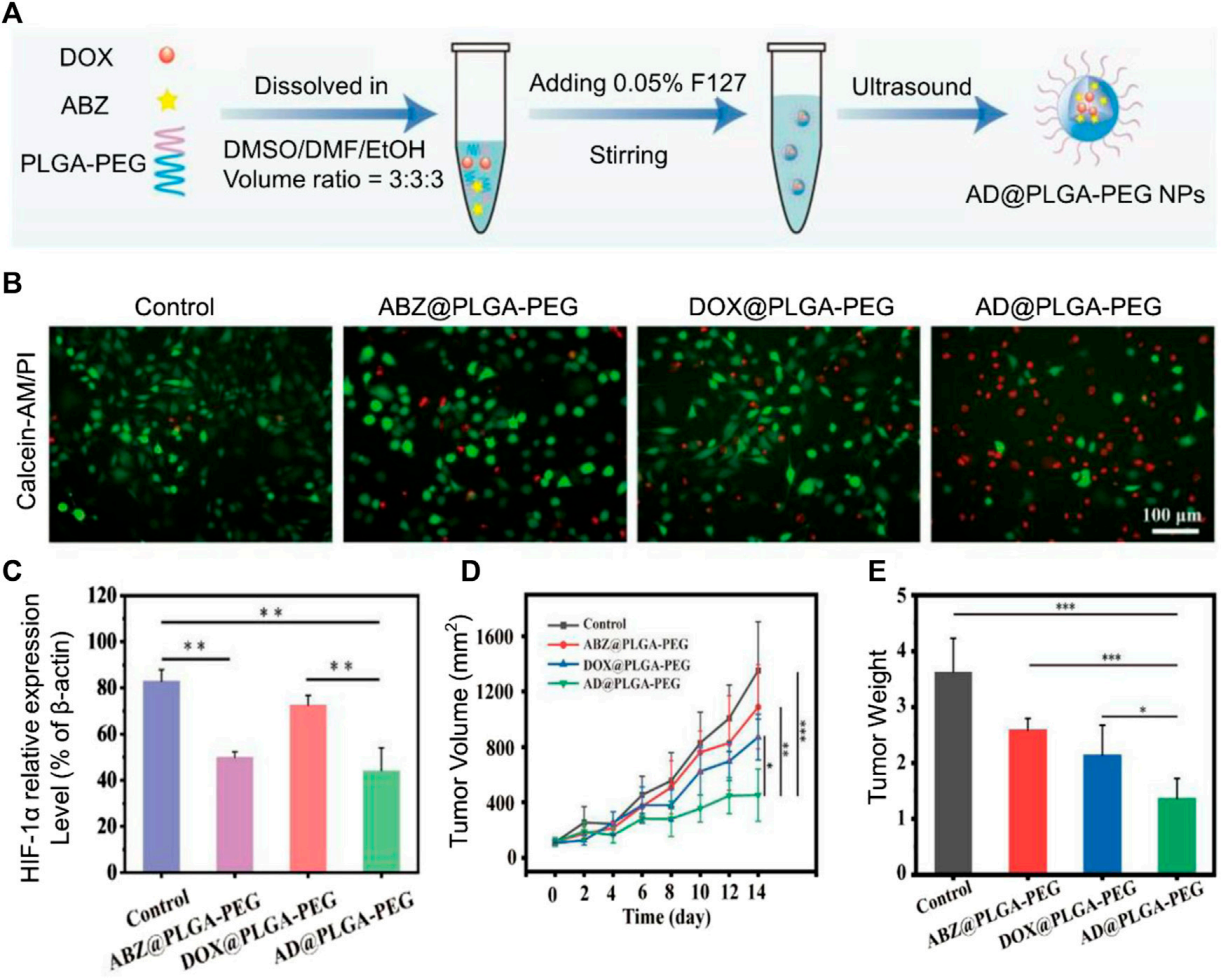
**(A)** AD@PLGA-PEG NPs production flowchart. **(B)** Antitumor activity of AD@PLGA-PEG NPs (red: dead cells, green: live cells). **(C)** Quantitative analysis of HIF-1α-related protein expression. **(D)** Tumor growth curve and tumor volume. **(E)** Tumor weights in different treatment groups. Reproduced with permission from (Zhao T.-T. et al., 2023a).

It is well known that in a hypoxic environment of the tumor microenvironment, the increase in oxygen is not conducive to tumor cell growth (Xia et al., 2022a). Hypoxia-inducible factor-1α (HIF-1α) is a hypoxia-stable transcription factor that regulates the expression of multiple target genes involved in glycolysis, mitochondrial respiration, tumor metastasis, and chemotherapy resistance (Akman et al., 2021). Strategies to alleviate hypoxia and inhibit HIF-1α in the tumor microenvironment are an important way to treat tumors (Niu et al., 2021). ROS acts as a double-edged sword for tumor cells (Jia et al., 2022). ROS also has different effects on cells because of different concentrations. The cause of tumor cells is that genes are mutated in the process of cell division and proliferation, and ROS will promote this process. The cause of cell death is the damage to cellular DNA and proteins caused by high concentrations of ROS (Wang et al., 2020a; Zheng et al., 2023). Albendazole (ABZ) is a safe HIF-1α inhibitor with low toxicity that has been approved by the FDA (Pourgholami et al., 2010). However, the low bioavailability and low solubility of ABZ limit its clinical application (García et al., 2014). Poly(lactic-co-glycolic acid)-polyethylene glycol (PLGA-PEG) nanoparticles (NPs) have been widely used in nanomedicine and regenerative medicine due to their biocompatibility (Xia et al., 2022b; Xia et al., 2023). Zhao et al. (2023a) fabricated AD@PLGA-PEG NPs with PLGA–PEG loads ABZ and DOX (ABZ and DOX molar ratio of 4:1) (Figure 2A). ABZ combined with DOX increases intracellular ROS and induces more tumor cell apoptosis compared to free DOX (Figure 2B) (Baldini et al., 1992). The particle size of AD@PLGA-PEG NP is about 140.51 ± 1.3 nm. When AD@PLGA-PEG NP is taken up by tumor cells, ABZ interferes with the mitochondrial respiratory chain, promotes oxidative stress, causes downregulation of HIF-related gene expression, and causes ROS production (Figure 2C). Compared with the control group, tumor volume was detected in tumor-bearing mice for 42 days (Figure 2D), the tumor size of the AD@PLGA-PEG NP group shrank threefold, and the tumor weight was also the lightest (Figure 2E). Not only that, AD@PLGA-PEG NP can also reduce the metastasis rate of OS.

Metal oxides are a material commonly used in biomedicine. Zirconia (ZrO2) has a strong ultraviolet absorption capacity and is widely used as a catalyst and photosensitizer (Zhang et al., 2021). Chianese et al. (2022) designed photoemissive ZrO2-acetylacetonate nanoparticles (ZrO2-acac NPs). Modifying hyaluronic acid on the surface of ZrO2-acac NPs can enhance the uptake of nanoparticles by tumor cells. ZrO2-acac NPs absorbed by tumor cells can release ZrO2, which can produce a large amount of ROS under light conditions, which in turn kills tumor cells. It was found that the particle size of ZrO2-acac NPs was 365 nm, and the loading rate of ZrO2 was 7.3% ± 0.1%. ZrO2-acac NPs and tumor cells were incubated for 48 h, and it was observed that ZrO2-acac NPs caused more tumor cell death than the control group. Zinc oxide nanoparticles (ZnO NPs) are widely used in the biomedical field, and their antibacterial, hemostatic, and other effects have been widely known (Shi et al., 2014). In recent years, the anti-tumor effect of ZnO NPs has gradually been well known. ZnO NPs have been reported to induce degradation of β-catenin mediated by HIF-1α/BNIP1/LC3B, triggering HIF-3 and Wnt pathway activation to inhibit OS cell metastasis (Zhang and Wang, 2020; He et al., 2023). Manganese dioxide (MnO2) can catalyze the decomposition of endogenous hydrogen peroxide in tumors to produce oxygen, thereby alleviating the hypoxic conditions of the tumor microenvironment (Liu et al., 2020). Phytic acid (PA) is a natural compound extracted from plants with certain antitumor activity, antioxidant, chelating agent and good bone targeting ability (Chen et al., 2018). Ju et al. (2023) made MnO2@PA NPs by modifying MnO2 NPs with PA. MnO2@PA NPs have a particle size of 111.1 ± 1.9 nm. *In vivo*, experiments confirmed that mice treated with MnO2@PA NPs had significantly smaller tumor volumes than controls within 16 days.

## 4 Mitochondrial autophagy nanoparticles

Autophagy is a highly conserved process in cellular catabolism. The effect of autophagy on cells is bidirectional, with proper levels of autophagy protecting cells from damage, while excessive or reduced autophagy can trigger apoptosis (Chen et al., 2015). Mitochondria, as the main site of adenosine triphosphate (ATP) supply, are the main source of cellular energy (Duan and Fang, 2016). Automitochondrial phagogy helps cells remove aging proteins and maintain the stability of mitochondrial structure and function. However, pathological mitochondrial autophagy can lead to structural disturbances of cells, and abnormal cell metabolism leads to cell death (Wang et al., 2020b). Exogenous induction of intracellular mitophagy to promote tumor cell necrosis has attracted attention in recent years (Sun et al., 2021). Hydroxyapatite nanoparticles (HANPs) can promote autophagy of tumor cells to cause anti-tumor activity (Li et al., 2020). This mitophagy is often associated with calcium levels within tumor cells. Calcium ions as a second signal, and its disorder can lead to the imbalance of cellular metabolism (Yin et al., 2021). Not only that, HANPs also promote bone regeneration. Wu et al. (2022b) designed HANPs of different sizes to assess the antitumor activity of HANPs. Studies have shown that HANPs can cause upregulation of mitochondrial apoptosis-related genes (p53, Bcl-2 and caspase proteins). The anti-inflammatory and antitumor properties of gallic acid (GA) have been widely demonstrated (Zamudio-Cuevas et al., 2021). GA has been reported to inhibit highly expressed heat shock proteins in tumor cells, affecting ATP production (Ding et al., 2022). Liu et al. (2023) coordinated metal ions (Fe and Mn) with GA to make nanoparticles that can release GA and metal ions on demand. The average size of FeGA and MnGA is 105.7 and 164.2 nm, respectively. Fe and Mn catalyze the Fenton reaction in cancer cells, leading to organelle damage. FeGA and MnGA cause changes in mitochondrial membrane potential (MMP), and a large amount of ROS enters tumor cells to cause mitochondrial dysfunction. Studies have shown that metallo-gallic acid nanoparticles first upregulate the mitochondrial apoptosis-related gene, Bax, thereby activating caspase-3 by upregulated intracellular ROS. In tumor-bearing mice, MnGA was enriched at the tumor site, with a targeting efficiency of 39.8% at 14 h, and no obvious tissue damage occurred in the heart, liver, spleen, lung, and kidney. This indicates that the amount of metal-GA nanoparticles recruited at the tumor site is greatly increased without causing significant adverse effects.

Exosomes (EVs) are a heterogeneous population of membrane vesicles secreted by all living cells. EVs represent a new form of cell-to-cell communication that promotes cell proliferation, and differentiation, and inhibits apoptosis and inflammation to mediate tissue repair by delivering the proteins, RNA, and lipids it carries to target cells (Xia et al., 2022c). Rifampicin (RIF) binds to the β subunit of RNA polymerase to achieve its antibacterial effect (Maggi et al., 2009). RIF is known for being a traditional anti-TB drug. It has been found that RIF can cause mitochondrial lysis, induce apoptosis and cell cycle arrest, and achieve its anti-tumor effect (Mieras et al., 2016). Chen et al. (2022) isolated bone marrow-derived mesenchymal stem cells by ultracentrifugation to generate EVs for RIF delivery to make EXO-RIFs. The particle size of EXO-RIF is 65–225 nm, and the drug release rate of EXO-RIF is found to be significantly higher at pH 4.5 than at pH 7.4. Since the membrane structure of EV is similar to that of tumor cells, it is conducive to the uptake of EXO-RIF by tumor cells. In tumor-bearing mice, the EXO-RIF treatment group showed decreased Ki67 expression, elevated c-caspase-3 and Bax, and low Bcl-2 expression compared to RIF treatment. Not only that, the ALT, AST, BUN, CR, CK, and CK-MB levels in the EXO-RIF treatment group were lower than in the RIF group. This result shows that EXO-RIF greatly reduces the systemic toxicity of RIF.

## 5 Nanoparticles induce bone regeneration

The destruction of bone by OS is one of the factors that cannot be ignored. Whether it is the damage to the bone by surgery or the damage to the bone by the OS itself, it brings serious harm to the patient. Not only that, but chemotherapy drugs can also cause damage to the bone microenvironment, destroying bone stability (Lamora et al., 2016). Being able to cause bone remodeling during the treatment of OS is considered a better strategy. miR-29b is a non-coding small RNA that regulates gene expression and has been shown *in vivo* experiments to inhibit proliferation and migration and induce apoptosis in OS cells (Chen et al., 2017). miR-29b can also significantly inhibit BCL-2 expression and upregulate Bax expression, promoting tumor cell expression. Poly β aminoester (pBAE) nanoparticles have repeating ester groups, are capable of degradation in water, and have low toxicity and high biocompatibility (Dosta et al., 2021). Freeman et al. (2023) developed a pBAE nanoparticle delivery vehicle for delivery of miR-29b to OS cells and surrounding stromal cells. Subsequently, Fiona et al. developed HA hydrogels for the sustained release of bone morphogenetic protein-2 (BMP-2) and pBAE nanoparticles. BMP-2 is a strong inducer of bone remodeling and promotes bone regeneration (Xia et al., 2022d). *In vitro*, experiments have shown that the HA hydrogel system can continuously release 18% of pBAE nanoparticles within 100 days and 42% of BMP-2 within 33 days. The pBAE nanoparticles are 151 ± 2 nm with a surface charge of 5.6 ± 3.5 mV. The released miR-29b inhibited tumor cell growth, and in tumor-bearing mice, pBAE nanoparticles reduced tumor volume by 15% within 45 days of treatment compared to the control group. Not only that, BAM-2 released by the HA hydrogel system can promote bone regeneration, and osteolysis is reduced by an average of 29% compared to the control group. Most importantly, HA hydrogel systems can greatly extend the continuous release capacity of pBAE nanoparticles.

**FIGURE 3:**
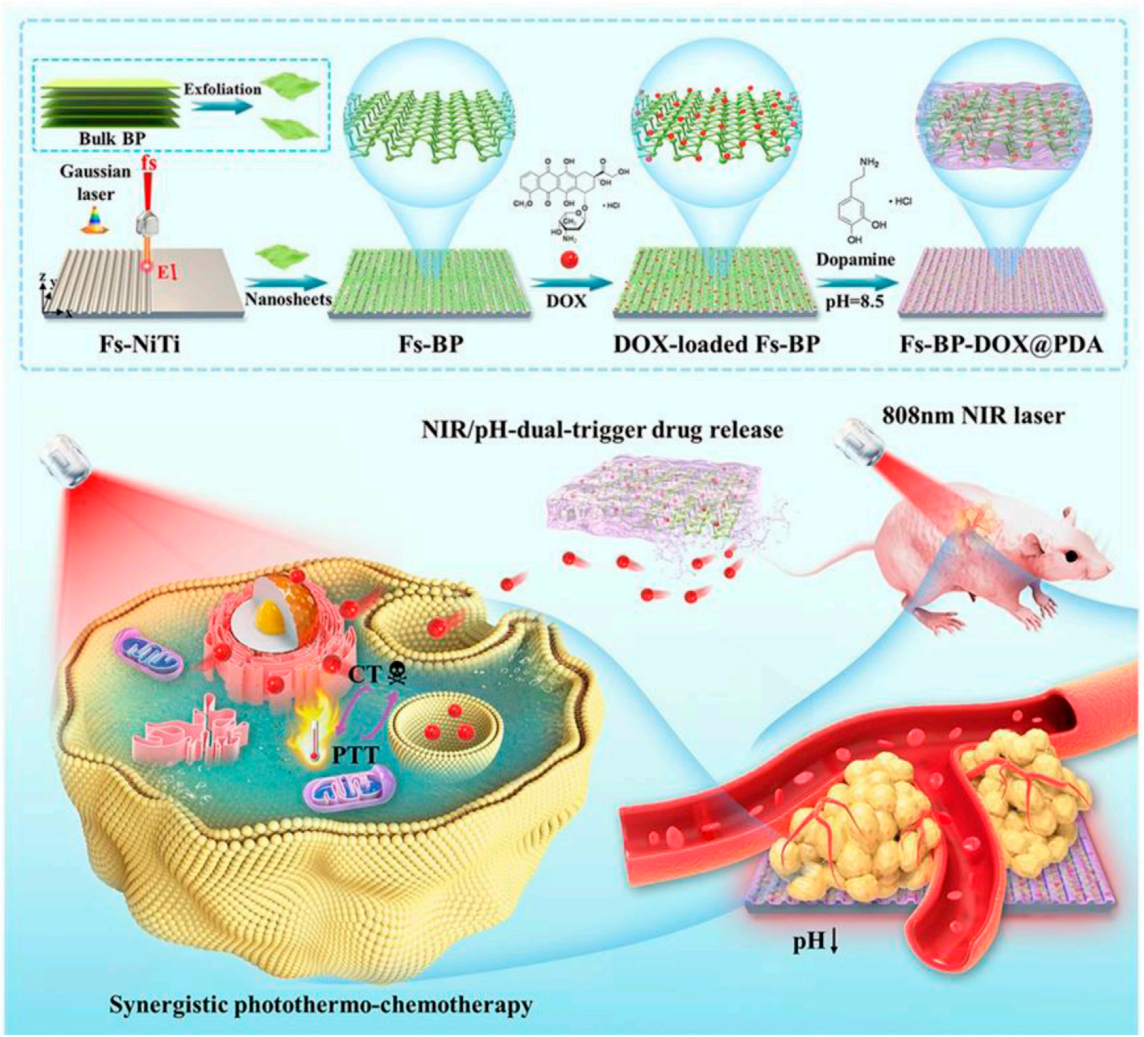
Schematic illustration of the design and fabrication strategy of the versatile multiscale therapeutic platform. Reproduced with permission from (Ma et al., 2022).

Ma et al. (2022) developed a fabricating groovelike micro-nanostructures (Fs-BP) using black phosphorus (BP) nanosheets for the delivery of DOX and PDA (Fs-BP-DOX@PDA) (Figure 3). PDA can enhance photothermal therapy of OS under near-infrared (NIR) mediation. The micro-nano layered structure of Fs-BP-DOX@PDA provides a strong surface area for bone regeneration. Fs-BP-DOX@PDA has load ratios of 93.2% and 35.2% for DOX and PDA, respectively. Fs-BP-DOX@PDA has higher photothermal conversion and stability under NIR. In tumor-bearing mice, Fs-BP-DOX@PDA was able to virtually eliminate tumor cells on day 14 and increase the survival rate of tumor-bearing mice to 60% after 100 days of treatment. What’s more, Fs-BP-DOX@PDA is able to release phosphate ions, which form calcium phosphate deposition with calcium ions around bone to promote osteoblast mineralization. FS-BP-DOX@PDA also has unique antibacterial properties, with clearances of 99.2% and 99.6% against *Staphylococcus aureus* and *Pseudomonas aeruginosa*, respectively. Fs-BP-DOX@PDA avoids poor bone repair due to bacterial infection.

Curcumin (CM) is a polyphenolic compound extracted from plants, and its anti-tumor properties are well known (Saleh et al., 2019; Lu et al., 2023). The low solubility and bioavailability of CM limit its clinical application. Silkfibroin (SF) has been well known in recent years for tumor treatment and bone regeneration (Asadpour et al., 2020). Han et al. (2023) developed SF/HA scaffolds using supercritical carbon dioxide (SC-CO2) technology, filling PDA-coated CM-loaded chitosan nanoparticles on SF/HA scaffolds to make CM-PDA/SF/nHA nanofibrous scaffolds. The average particle size of 204.7 nm for nanoparticles. The porosity of the stent is 77.3%. Large porosity and compressive strength can meet the conditions for bone regeneration. SF/HA stent is able to continuously release CM and SF around the tumor, and in the presence of PDA, can enhance photothermal therapy. In mice, CM-PDA/SF/nHA nanofibrous scaffolds were detected to cause an increase in alkaline phosphatase, an early marker of bone differentiation, at 40 days.

## 6 Nanoparticles for delivery strategies

Nanoparticles are widely used in nanomedicine because of their precise delivery and slow release of carriers. The surface of the nanoparticle can be modified with different groups to set up nanoparticles with different functions (Hoang et al., 2022). Nano-loaded drugs can reduce the toxic side effects of the drug itself and achieve precise release of the tumor site (Iinuma et al., 2002). Cisplatin (CDDP) is one of the commonly used chemotherapy drugs for OS, and its mechanism of action is to bind to DNA, destroy DNA and inhibit tumor cell mitosis (Dasari and Bernard Tchounwou, 2014; Xiang et al., 2023). However, CDDP has strong neurotoxicity and gastrointestinal reactions (Isakoff et al., 2015). Nanoliposomes are highly biocompatible drug-loading carriers. Alendronate can inhibit osteoclast activity and is considered a bone targeting vehicle (Fisher et al., 1999). Alendronate bindronate specifically binds to hydroxyapatite in the bone system for bone targeting. Zhong et al. (2023) made LCA NPs with sodium alendronate attached to liposomes loaded with CDDP. The particle size of LCA NPs is 144.4 ± 20.23 nm. *In vitro*, experiments have shown that LCA NPs can kill 99% of tumor cells. The encapsulation of nanoliposomes alleviates the systemic toxic side effects of CDDP. Wei et al. (2019) demonstrated anti-tumor effects *in vitro* with bone marrow-derived EVs-loaded DOX. The DOX-loaded EVs have a particle size of 152.7 nm. The vesicle structure of EVs allows them to easily bind to tumor cell membranes and release DOX into tumor cells. The encapsulation efficiency of the drug is about 7%, and the release of Exo-Dox after 60 h is about 36%. Compared to free Dox, Exo-Dox can kill OS cells more efficiently and exhibits lower cytotoxicity in cardiomyocytes. Ginsenosides can inhibit the growth of cancer cells, induce apoptosis of tumor cells and improve immunity (Han et al., 2006). However, ginsenosides have low bioavailability and are difficult to absorb by the body (Hasegawa et al., 1996). Fu et al. (2022) modified tumor cell membrane-camouflaged nanoparticles with alendronate sodium for delivery of MnO2 and ginsenosides. The particle size of the nanoparticles is 141.5 nm, and the nanoparticle seeds have good bone targeting ability due to the addition of sodium alendronate. MnO2 can promote the breakdown of H2O2 in the body and enhance tumor killing. The nanoparticles enhance immune cell infiltration and improve mouse survival up to 55 days.

**FIGURE 4:**
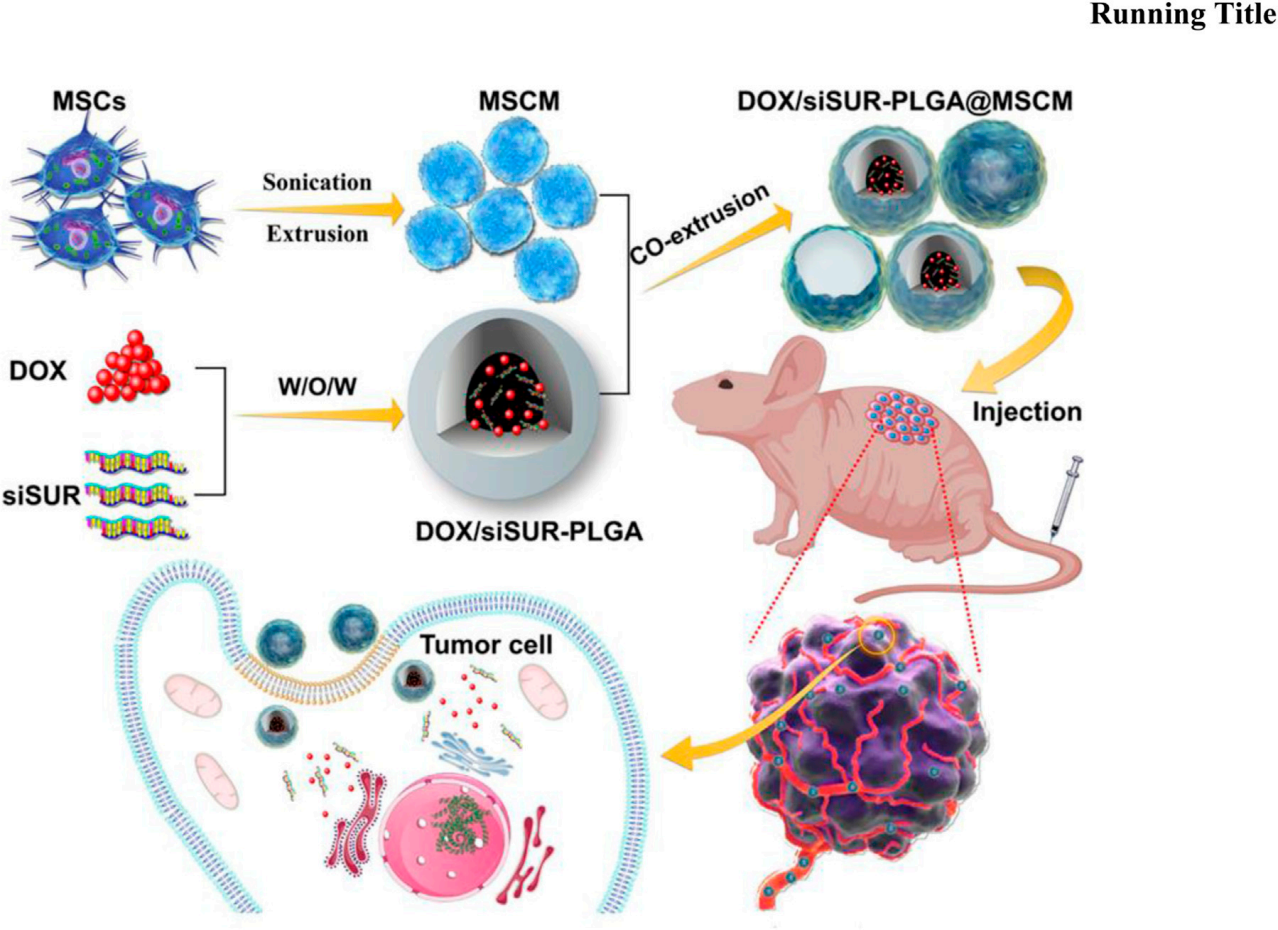
Mesenchymal stem cell membrane-camouflaged nanoparticles coloaded with DOX and survivin siRNA for osteosarcoma treatment. Reproduced with permission from (Zhao J. et al., 2023b).

Mesenchymal stem cells have unique tumor homing and migration effects and can be used for tumor targeting (Chen and Liu, 2016). Zhao et al. (2023b) used solvent evaporation to load DOX and siRNA into MSCMs-modified PLGAs to make DOX/siRNA-PLGA@MSCM NPs (Figure 4). The encapsulation rate and drug load of DOX/siRNA-PLGA@MSCM NPs were 53.10% ± 1.45% and 53.94 ± 2.31, respectively. *In vitro* experiments showed that DOX/siRNA-PLGA@MSCM NPs could be endocytosed by tumor cells within 3 h, and the cumulative release of DOX within 48 h was 26.67%. After 48 h of mouse tail vein injection of DOX/siRNA-PLGA@MSCM NPs, DOX/siRNA-PLGA@MSCM NPs were mainly deposited at the tumor site. This suggests that DOX/siRNA-PLGA@MSCM NPs have a strong ability to target tumor cells. The antitumor activity of DOX/siRNA-PLGA@MSCM NPs was detected in tumor-bearing mice, and DOX/siRNA-PLGA@MSCM NPs could promote apoptosis of tumor cells and reduce the metastasis rate of tumors. Not only that but DOX/siRNA-PLGA@MSCM NPs also greatly reduced the adverse events of cardiotoxicity and bone marrow suppression of DOX. Zoledronic acid (ZOL) has a significant effect in promoting apoptosis of tumor cells, inhibiting angiogenesis and metastasis, and is a bisphosphonate for the prevention and treatment of metastatic bone disease (Nadar et al., 2017; Wang et al., 2020c). Xu et al. (2022) used HA and PEG to synthesize nanoparticles to deliver ZOL to make HA-PEG-N-HA-ZOL NP. HA-PEG-NHA-ZOL NP has a particle size of 159 ± 2.3 nm.

HA-PEG-NHA-ZOL NP could increase the expression level of Bcl-2-associated X protein (BAX), indicating that HA-PG-NHA-ZOL NP could promote apoptosis in tumor cells. HA-PEG-NHA-ZOL NP was shown in mice to reduce the expression of tumor cells Ki-67 and inhibit tumor cell proliferation. In mice for 5 days, HA-PEG-NHA-ZOL NP did not cause lung, liver, spleen, kidney and other functions. AbouAitah et al. (2022) developed an example of a core-shell nanoparticle that uses mesoporous silica nanoparticles (MSN) loaded colchicine (CL) to make nanoparticle shells, and chitosan-curcumin mixtures to make nanoparticle nuclei. The high surface area, large size, and large pore structure of MSN favor its use as a carrier-loaded drug (AbouAitah et al., 2020). *In vivo* experimental results show that core-shell nanoparticles can improve the anti-cancer treatment efficiency of CL while reducing its toxicity to normal cells.

## 7 Conclusion

OS is highly malignant, highly aggressive and metastatic. Although chemotherapy improves survival in OS. However, the side effects of chemotherapy drugs and drug resistance in OS remain treatment challenges. Preclinical studies have demonstrated that nanoparticles are effective in delaying the growth of OS and improving the survival rate of OS patients. In recent years, several nanocarrier-based drug delivery systems have been explored to target and treat OS. Although the nano-delivery system has made breakthroughs in the treatment of OS, improving the survival rate of OS mice. However, most of the current research is limited to *in vitro* cell experiments and animal experiments, which is still far from clinical application. Appropriate nanoparticles need to meet the following conditions: 1) Nanoparticles can effectively target OS cells; 2) Nanoparticles are able to accumulate and release payloads in OS cells; 3) Nanoparticles are reduced in other organs (heart, liver, kidney, etc.) that are aggregated to reduce systemic complications 4) Nanoparticles have good biosafety and bioavailability. Based on the current research base, we believe that the treatment of OS by nanoparticles will achieve good clinical results.

## References

[B1] AbouAitah K. Hassan H. A. Swiderska-Sroda A. Gohar L. Shaker O. G. Wojnarowicz J. (2020). Targeted nano-drug delivery of colchicine against colon cancer cells by means of mesoporous silica nanoparticles. Cancers 12, 144. 10.3390/cancers12010144 31936103 PMC7017376

[B2] AbouAitah K. Soliman A. A. F. Swiderska-Sroda A. Nassrallah A. Smalc-Koziorowska J. Gierlotka S. (2022). Co-delivery system of curcumin and colchicine using functionalized mesoporous silica nanoparticles promotes anticancer and apoptosis effects. Pharmaceutics 14, 2770. 10.3390/pharmaceutics14122770 36559264 PMC9785757

[B3] Jemal A. Murray T. Ward E. Samuels A. Tiwari R. C. Ghafoor A. (2005). Cancer statistics, CA a cancer J. Clin. 55 10–30. 10.3322/canjclin.55.1.10 15661684

[B4] Akman M. Belisario D. C. Salaroglio I. C. Kopecka J. Donadelli M. De Smaele E. (2021). Hypoxia, endoplasmic reticulum stress and chemoresistance: dangerous liaisons. J. Exp. Clin. Cancer Res. 40, 28. 10.1186/s13046-020-01824-3 33423689 PMC7798239

[B5] Alfranca A. Martinez-Cruzado L. Tornin J. Abarrategi A. Amaral T. de Alava E. (2015). Bone microenvironment signals in osteosarcoma development. Cell. Mol. Life Sci. 72, 3097–3113. 10.1007/s00018-015-1918-y 25935149 PMC11113487

[B6] Asadpour S. Kargozar S. Moradi L. Ai A. Nosrati H. Ai J. (2020). Natural biomacromolecule based composite scaffolds from silk fibroin, gelatin and chitosan toward tissue engineering applications. Int. J. Biol. Macromol. 154, 1285–1294. 10.1016/j.ijbiomac.2019.11.003 31733251

[B7] Avnet S. Longhi A. Salerno M. Halleen J. M. Perut F. Granchi D. (2008). Increased osteoclast activity is associated with aggressiveness of osteosarcoma. Int. J. Oncol. 33, 1231–1238. 10.3892/ijo_00000113 19020756

[B8] Baldini N. Scotlandi K. Serra M. Kusuzaki K. Shikita T. Manara M. C. (1992). Adriamycin binding assay: A valuable chemosensitivity test in human osteosarcoma. J. Cancer Res. Clin. Oncol. 119, 121–126. 10.1007/BF01209668 1358894 PMC12200449

[B9] Chen F. M. Liu X. (2016). Advancing biomaterials of human origin for tissue engineering. Prog. Polym. Sci. 53, 86–168. 10.1016/j.progpolymsci.2015.02.004 27022202 PMC4808059

[B10] Chen H. X. Xu X. X. Tan B. Z. Zhang Z. Zhou X. D. (2017). MicroRNA-29b inhibits angiogenesis by targeting VEGFA through the MAPK/ERK and PI3K/akt signaling pathways in endometrial carcinoma. Cell. physiology Biochem. Int. J. Exp. Cell. physiology, Biochem. Pharmacol. 41, 933–946. 10.1159/000460510 28222438

[B11] Chen J.-W. Ni B.-B. Zheng X.-F. Li B. Jiang S.-D. Jiang L.-S. (2015). Hypoxia facilitates the survival of nucleus pulposus cells in serum deprivation by down-regulating excessive autophagy through restricting ROS generation. Int. J. Biochem. Cell. Biol. 59, 1–10. 10.1016/j.biocel.2014.11.009 25456445

[B12] Chen K. Zhang S. Li A. Tang X. Li L. Guo L. (2018). Bioinspired interfacial chelating-like reinforcement strategy toward mechanically enhanced lamellar materials. ACS Nano 12, 4269–4279. 10.1021/acsnano.7b08671 29697956

[B13] Chen W. Lin W. Yu N. Zhang L. Wu Z. Chen Y. (2022). Activation of dynamin-related protein 1 and induction of mitochondrial apoptosis by exosome-rifampicin nanoparticles exerts anti-osteosarcoma effect. Int. J. nanomedicine 17, 5431–5446. 10.2147/IJN.S379917 36426375 PMC9680970

[B14] Chianese G. Fasolino I. Tramontano C. De Stefano L. Imparato C. Aronne A. (2022). ROS-generating hyaluronic acid-modified zirconium dioxide-acetylacetonate nanoparticles as a theranostic platform for the treatment of osteosarcoma. Nanomater. (Basel, Switz. 13, 54. 10.3390/nano13010054 PMC9823868 36615964

[B15] Corre I. Verrecchia F. Crenn V. Redini F. Trichet V. (2020). The osteosarcoma microenvironment: A complex but targetable ecosystem. Cells 9, 976. 10.3390/cells9040976 32326444 PMC7226971

[B16] Dasari S. Bernard Tchounwou P. (2014). Cisplatin in cancer therapy: molecular mechanisms of action. Eur. J. Pharmacol. 740, 364–378. 10.1016/j.ejphar.2014.07.025 25058905 PMC4146684

[B17] Deng J. Liu S. Li G. Zheng Y. Zhang W. Lin J. (2023). pH-sensitive charge-conversion cinnamaldehyde polymeric prodrug micelles for effective targeted chemotherapy of osteosarcoma *in vitro* . Front. Chem. 11, 1190596. 10.3389/fchem.2023.1190596 37206197 PMC10188981

[B18] Ding X.-L. Liu M.-D. Cheng Q. Guo W.-H. Niu M.-T. Huang Q.-X. (2022). Multifunctional liquid metal-based nanoparticles with glycolysis and mitochondrial metabolism inhibition for tumor photothermal therapy. Biomaterials 281, 121369. 10.1016/j.biomaterials.2022.121369 35026671

[B19] Dosta P. Tamargo I. Ramos V. Kumar S. Kang D. W. Borrós S. (2021). Delivery of anti-microRNA-712 to inflamed endothelial cells using poly(β-amino ester) nanoparticles conjugated with VCAM-1 targeting peptide. Adv. Healthc. Mater. 10, e2001894. 10.1002/adhm.202001894 33448151 PMC8277885

[B20] Duan Y. Fang H. (2016). RecQL4 regulates autophagy and apoptosis in U2OS cells. Biochem. Cell. Biol. 94, 551–559. 10.1139/bcb-2016-0005 27813658

[B21] Eaton B. R. Schwarz R. Vatner R. Yeh B. Claude L. Indelicato D. J. (2021). Osteosarcoma. Pediatr. Blood Cancer 68, e28352. 10.1002/pbc.28352 32779875

[B22] Feng Z. Guo J. Liu X. Song H. Zhang C. Huang P. (2020). Cascade of reactive oxygen species generation by polyprodrug for combinational photodynamic therapy. Biomaterials 255, 120210. 10.1016/j.biomaterials.2020.120210 32592871

[B23] Fisher J. E. Rogers M. J. Halasy J. M. Luckman S. P. Hughes D. E. Masarachia P. J. (1999). Alendronate mechanism of action: geranylgeraniol, an intermediate in the mevalonate pathway, prevents inhibition of osteoclast formation, bone resorption, and kinase activation *in vitro* . Proc. Natl. Acad. Sci. 96, 133–138. 10.1073/pnas.96.1.133 9874784 PMC15105

[B24] Freeman F. E. Dosta P. Shanley L. C. Ramirez Tamez N. Riojas Javelly C. J. Mahon O. R. (2023). Localized nanoparticle-mediated delivery of miR-29b normalizes the dysregulation of bone homeostasis caused by osteosarcoma whilst simultaneously inhibiting tumor growth. Adv. Mater. 35, 2207877. 10.1002/adma.202207877 36994935

[B25] Fu L. Zhang W. Zhou X. Fu J. He C. (2022). Tumor cell membrane-camouflaged responsive nanoparticles enable MRI-guided immuno-chemodynamic therapy of orthotopic osteosarcoma. Bioact. Mater. 17, 221–233. 10.1016/j.bioactmat.2022.01.035 35386464 PMC8965157

[B26] García A. Leonardi D. Salazar M. O. Lamas M. C. (2014). Modified β-cyclodextrin inclusion complex to improve the physicochemical properties of albendazole. complete *in vitro* evaluation and characterization. PloS one 9, e88234. 10.1371/journal.pone.0088234 24551084 PMC3925136

[B27] Han J. Y. Kwon Y. S. Yang D. C. Jung Y. R. Choi Y. E. (2006). Expression and RNA interference-induced silencing of the dammarenediol synthase gene in Panax ginseng. Plant & Cell. physiology 47, 1653–1662. 10.1093/pcp/pcl032 17088293

[B28] Han R. Min Y. Li G. Chen S. Xie M. Zhao Z. (2023). Supercritical CO(2)-assisted fabrication of CM-PDA/SF/nHA nanofibrous scaffolds for bone regeneration and chemo-photothermal therapy against osteosarcoma. Biomaterials Sci. 11, 5218–5231. 10.1039/d3bm00532a 37338001

[B29] Hasegawa H. Sung J. H. Matsumiya S. Uchiyama M. (1996). Main ginseng saponin metabolites formed by intestinal bacteria. Planta medica. 62, 453–457. 10.1055/s-2006-957938 8923812

[B30] He G. Nie J. J. Liu X. Ding Z. Luo P. Liu Y. (2023). Zinc oxide nanoparticles inhibit osteosarcoma metastasis by downregulating β-catenin via HIF-1α/BNIP3/LC3B-mediated mitophagy pathway. Bioact. Mater. 19, 690–702. 10.1016/j.bioactmat.2022.05.006 35600978 PMC9112061

[B31] Hoang K. N. L. McClain S. M. Meyer S. M. Jalomo C. A. Forney N. B. Murphy C. J. (2022). Site-selective modification of metallic nanoparticles. Chem. Commun. Camb. Engl. 58, 9728–9741. 10.1039/d2cc03603g 35975479

[B32] Hu D. Deng Y. Jia F. Jin Q. Ji J. (2020). Surface charge switchable supramolecular nanocarriers for nitric oxide synergistic photodynamic eradication of biofilms. ACS Nano 14, 347–359. 10.1021/acsnano.9b05493 31887012

[B33] Huang Y. Chen J. Yang S. Tan T. Wang N. Wang Y. (2020). Cinnamaldehyde inhibits the function of osteosarcoma by suppressing the wnt/β-catenin and PI3K/akt signaling pathways. Drug Des. Dev. Ther. 14, 4625–4637. 10.2147/DDDT.S277160 PMC7608596 33154629

[B34] Iinuma H. Maruyama K. Okinaga K. Sasaki K. Sekine T. Ishida O. (2002). Intracellular targeting therapy of cisplatin-encapsulated transferrin-polyethylene glycol liposome on peritoneal dissemination of gastric cancer. Int. J. Cancer 99, 130–137. 10.1002/ijc.10242 11948504

[B35] Isakoff M. S. Bielack S. S. Meltzer P. Gorlick R. (2015). Osteosarcoma: current treatment and a collaborative pathway to success. J. Clin. Oncol. 33, 3029–3035. 10.1200/JCO.2014.59.4895 26304877 PMC4979196

[B36] Jia F. Liu Y. Dou X. Du C. Mao T. Liu X. (2022). Liensinine inhibits osteosarcoma growth by ROS-mediated suppression of the JAK2/STAT3 signaling pathway. Oxidative Med. Cell. Longev. 2022, 8245614. 10.1155/2022/8245614 PMC8807040 35116094

[B37] Ju Q. Huang R. Hu R. Fan J. Zhang D. Ding J. (2023). Phytic acid-modified manganese dioxide nanoparticles oligomer for magnetic resonance imaging and targeting therapy of osteosarcoma. Drug Deliv. 30, 2181743. 10.1080/10717544.2023.2181743 36855959 PMC9980014

[B38] Lamora A. Talbot J. Mullard M. Brounais-Le Royer B. Redini F. Verrecchia F. (2016). TGF-Β signaling in bone remodeling and osteosarcoma progression. J. Clin. Med. 5, 96. 10.3390/jcm5110096 27827889 PMC5126793

[B39] Li S. Zhang H. Liu J. Shang G. (2023). Targeted therapy for osteosarcoma: A review. J. Cancer Res. Clin. Oncol. 149, 6785–6797. 10.1007/s00432-023-04614-4 36807762 PMC11796608

[B40] Li X. Wang Y. Chen Y. Zhou P. Wei K. Wang H. (2020). Hierarchically constructed selenium-doped bone-mimetic nanoparticles promote ROS-mediated autophagy and apoptosis for bone tumor inhibition. Biomaterials 257, 120253. 10.1016/j.biomaterials.2020.120253 32738660

[B41] Liu J. Zhang W. Kumar A. Rong X. Yang W. Chen H. (2020). Acridine orange encapsulated mesoporous manganese dioxide nanoparticles to enhance radiotherapy. Bioconjugate Chem. 31, 82–92. 10.1021/acs.bioconjchem.9b00751 31809019

[B42] Liu K. Zhang L. Lu H. Wen Y. Bi B. Wang G. (2023). Enhanced mild-temperature photothermal therapy by pyroptosis-boosted ATP deprivation with biodegradable nanoformulation. J. nanobiotechnology 21, 64. 10.1186/s12951-023-01818-1 36823540 PMC9948333

[B43] Lu K. H. Lu P. W. Lin C. W. Yang S. F. (2023). Curcumin in human osteosarcoma: from analogs to carriers. Drug Discov. today 28, 103437. 10.1016/j.drudis.2022.103437 36372327

[B44] Ma Y. Jiang L. Hu J. Zhu E. Zhang N. (2022). Developing a versatile multiscale therapeutic platform for osteosarcoma synergistic photothermo-chemotherapy with effective osteogenicity and antibacterial capability. ACS Appl. Mater. Interfaces 14, 44065–44083. 10.1021/acsami.2c10772 36125961

[B45] Maggi N. Pasqualucci C. R. Ballotta R. Sensi P. (2009). Rifampicin: A new orally active rifamycin. Chemotherapia 11, 285–292. 10.1159/000220462 5958716

[B46] Maiuri M. C. Zalckvar E. Kimchi A. Kroemer G. (2007). Self-eating and self-killing: crosstalk between autophagy and apoptosis. Nat. Rev. Mol. Cell. Biol. 8, 741–752. 10.1038/nrm2239 17717517

[B47] Mieras L. Anthony R. van Brakel W. Bratschi M. W. van den Broek J. Cambau E. (2016). Negligible risk of inducing resistance in *Mycobacterium tuberculosis* with single-dose rifampicin as post-exposure prophylaxis for leprosy. Infect. Dis. Poverty 5, 46. 10.1186/s40249-016-0140-y 27268059 PMC4897814

[B48] Morales-Orue I. Chicas-Sett R. Lara P. C. (2019). Nanoparticles as a promising method to enhance the abscopal effect in the era of new targeted therapies. Rep. Pract. Oncol. Radiotherapy 24, 86–91. 10.1016/j.rpor.2018.11.001 PMC6251334 30505238

[B49] Nadar R. A. Margiotta N. Iafisco M. van den Beucken J. J. J. P. Boerman O. C. Leeuwenburgh S. C. G. (2017). Bisphosphonate-Functionalized imaging agents, anti-tumor agents and nanocarriers for treatment of bone cancer. Adv. Healthc. Mater. 6, 1601119. 10.1002/adhm.201601119 28207199

[B50] Nguyen T. L. Cha B. G. Choi Y. Im J. Kim J. (2020). Injectable dual-scale mesoporous silica cancer vaccine enabling efficient delivery of antigen/adjuvant-loaded nanoparticles to dendritic cells recruited in local macroporous scaffold. Biomaterials 239, 119859. 10.1016/j.biomaterials.2020.119859 32070828

[B51] Niu G. Zhou M. Wang F. Yang J. Huang J. Zhu Z. (2021). Marein ameliorates Ang II/hypoxia‐induced abnormal glucolipid metabolism by modulating the HIF ‐1α/PPARα/γ pathway in H9c2 cells. Drug Dev. Res. 82, 523–532. 10.1002/ddr.21770 33314222

[B52] Pourgholami M. H. Khachigian L. M. Fahmy R. G. Badar S. Wang L. Chu S. W. L. (2010). Albendazole inhibits endothelial cell migration, tube formation, vasopermeability, VEGF receptor-2 expression and suppresses retinal neovascularization in ROP model of angiogenesis. Biochem. Biophysical Res. Commun. 397, 729–734. 10.1016/j.bbrc.2010.06.019 20537982

[B53] Saleh T. Soudi T. Shojaosadati S. A. (2019). Aptamer functionalized curcumin-loaded human serum albumin (HSA) nanoparticles for targeted delivery to HER-2 positive breast cancer cells. Int. J. Biol. Macromol. 130, 109–116. 10.1016/j.ijbiomac.2019.02.129 30802519

[B54] Sergi C. M. (2021). Targeting the 'garbage-bin' to fight cancer: HDAC6 inhibitor WT161 has an anti-tumor effect on osteosarcoma and synergistically interacts with 5-FU. Biosci. Rep. 41. 10.1042/BSR20210952 PMC8350430 34323266

[B55] Shao R. Wang Y. Li L. Dong Y. Zhao J. Liang W. (2022). Bone tumors effective therapy through functionalized hydrogels: current developments and future expectations. Drug Deliv. 29, 1631–1647. 10.1080/10717544.2022.2075983 35612368 PMC9154780

[B56] Shi L. E. Li Z. H. Zheng W. Zhao Y. F. Jin Y. F. Tang Z. X. (2014). Synthesis, antibacterial activity, antibacterial mechanism and food applications of ZnO nanoparticles: A review. Food Addit. Contam. Part A, Chem. analysis, control, Expo. risk Assess. 31, 173–186. 10.1080/19440049.2013.865147 24219062

[B57] Shi Q. Lu Y. Zhang G. Yang X. Li R. Zhang G. (2022). Multifunctional mesoporous silica nanoparticles for pH-response and photothermy enhanced osteosarcoma therapy. Colloids Surfaces B Biointerfaces 217, 112615. 10.1016/j.colsurfb.2022.112615 35759893

[B58] Siclari V. A. Qin L. (2010). Targeting the osteosarcoma cancer stem cell. J. Orthop. Surg. Res. 5, 78. 10.1186/1749-799X-5-78 20979639 PMC2988747

[B59] Somaiah N. Conley A. P. Parra E. R. Lin H. Amini B. Solis Soto L. (2022). Durvalumab plus tremelimumab in advanced or metastatic soft tissue and bone sarcomas: A single-centre phase 2 trial. Lancet. Oncol. 23, 1156–1166. 10.1016/s1470-2045(22)00392-8 35934010

[B60] Sun K. Jing X. Guo J. Yao X. Guo F. (2021). Mitophagy in degenerative joint diseases. Autophagy 17, 2082–2092. 10.1080/15548627.2020.1822097 32967533 PMC8496714

[B61] Tarn D. Ashley C. E. Xue M. Carnes E. C. Zink J. I. Brinker C. J. (2013). Mesoporous silica nanoparticle nanocarriers: biofunctionality and biocompatibility. Accounts Chem. Res. 46, 792–801. 10.1021/ar3000986 PMC3686852 23387478

[B62] von Schacky C. E. Wilhelm N. J. Schäfer V. S. Leonhardt Y. Jung M. Jungmann P. M. (2022). Development and evaluation of machine learning models based on X-ray radiomics for the classification and differentiation of malignant and benign bone tumors. Eur. Radiol. 32, 6247–6257. 10.1007/s00330-022-08764-w 35396665 PMC9381439

[B63] Wang L. Fang D. Xu J. Luo R. (2020c). Various pathways of zoledronic acid against osteoclasts and bone cancer metastasis: A brief review. BMC Cancer 20, 1059. 10.1186/s12885-020-07568-9 33143662 PMC7607850

[B64] Wang S. Deng Z. Ma Y. Jin J. Qi F. Li S. (2020b). The role of autophagy and mitophagy in bone metabolic disorders. Int. J. Biol. Sci. 16, 2675–2691. 10.7150/ijbs.46627 32792864 PMC7415419

[B65] Wang S. Li H. Chen S. Wang Z. Yao Y. Chen T. (2020a). Andrographolide induces apoptosis in human osteosarcoma cells via the ROS/JNK pathway. Int. J. Oncol. 56, 1417–1428. 10.3892/ijo.2020.5032 32236589 PMC7170044

[B66] Wei H. Chen J. Wang S. Fu F. Zhu X. Wu C. (2019). A nanodrug consisting of doxorubicin and exosome derived from mesenchymal stem cells for osteosarcoma treatment *in vitro* . Int. J. nanomedicine 14, 8603–8610. 10.2147/IJN.S218988 31802872 PMC6830377

[B67] Wu H. Liu S. Chen S. Hua Y. Li X. Zeng Q. (2022b). A selective reduction of osteosarcoma by mitochondrial apoptosis using hydroxyapatite nanoparticles. Int. J. nanomedicine 17, 3691–3710. 10.2147/IJN.S375950 36046839 PMC9423115

[B68] Wu W. Guo H. Jing D. Zhang Z. Zhang Z. Pu F. (2022a). Targeted delivery of PD-L1-derived phosphorylation-mimicking peptides by engineered biomimetic nanovesicles to enhance osteosarcoma treatment. Adv. Healthc. Mater. 11, e2200955. 10.1002/adhm.202200955 36123781 PMC11468027

[B69] Xia Y. Wang H. Li Y. Fu C. (2022d). Engineered bone cement trigger bone defect regeneration, 9.

[B70] Xia Y. Wang H. Yang R. Hou Y. Li Y. Zhu J. (2023). Biomaterials delivery strategies to repair degenerated intervertebral discs by regulating the inflammatory microenvironment. Front. Immunol. 14, 1051606. 10.3389/fimmu.2023.1051606 36756124 PMC9900107

[B71] Xia Y. Yang R. Hou Y. Wang H. Li Y. Zhu J. (2022c). Application of mesenchymal stem cell-derived exosomes from different sources in intervertebral disc degeneration. Front. Bioeng. Biotechnol. 10, 1019437. 10.3389/fbioe.2022.1019437 36277386 PMC9585200

[B72] Xia Y. Yang R. Wang H. Hou Y. Li Y. Zhu J. (2022b). Biomaterials delivery strategies to repair spinal cord injury by modulating macrophage phenotypes. J. tissue Eng. 13, 20417314221143059. 10.1177/20417314221143059 36600997 PMC9806413

[B73] Xia Y. Yang R. Zhu J. Wang H. Li Y. Fan J. (2022a). Engineered nanomaterials trigger abscopal effect in immunotherapy of metastatic cancers. Front. Bioeng. Biotechnol. 10, 890257. 10.3389/fbioe.2022.890257 36394039 PMC9643844

[B74] Xiang D. Han X. Li J. Zhang J. Xiao H. Li T. (2023). Combination of Ido inhibitors and platinum(IV) prodrugs reverses low immune responses to enhance cancer chemotherapy and immunotherapy for osteosarcoma. Mater. today Bio 20, 100675. 10.1016/j.mtbio.2023.100675 PMC10250924 37304579

[B75] Xu Y. Qi J. Sun W. Zhong W. Wu H. (2022). Therapeutic effects of zoledronic acid-loaded hyaluronic acid/polyethylene glycol/nano-hydroxyapatite nanoparticles on osteosarcoma. Front. Bioeng. Biotechnol. 10, 897641. 10.3389/fbioe.2022.897641 35694235 PMC9181619

[B76] Yang F. Wen X. Ke Q.-F. Xie X.-T. Guo Y.-P. (2018). pH-responsive mesoporous ZSM-5 zeolites/chitosan core-shell nanodisks loaded with doxorubicin against osteosarcoma. Mater. Sci. Eng. C 85, 142–153. 10.1016/j.msec.2017.12.024 29407142

[B77] Yin Y. Jiang T. Hao Y. Zhang J. Li W. Hao Y. (2021). Cascade catalytic nanoplatform based on ions interference strategy for calcium overload therapy and ferroptosis. Int. J. Pharm. 606, 120937. 10.1016/j.ijpharm.2021.120937 34310960

[B78] Yuan P. Min Y. Zhao Z. (2023). Multifunctional nanoparticles for the treatment and diagnosis of osteosarcoma. Biomater. Adv. 151, 213466. 10.1016/j.bioadv.2023.213466 37229927

[B79] Zamudio-Cuevas Y. Andonegui-Elguera M. A. Aparicio-Juárez A. Aguillón-Solís E. Martínez-Flores K. Ruvalcaba-Paredes E. (2021). The enzymatic poly(gallic acid) reduces pro-inflammatory cytokines *in vitro*, a potential application in inflammatory diseases. Inflammation 44, 174–185. 10.1007/s10753-020-01319-5 32803665

[B80] Zhang C. Guo X. Xu Y. Han X. Cai J. Wang X. (2019). Lung metastases at the initial diagnosis of high-grade osteosarcoma: prevalence, risk factors and prognostic factors. A large population-based cohort study. Sao Paulo Med. J. = Revista paulista de Med. 137, 423–429. 10.1590/1516-3180.2018.0381120619 PMC9745822 31939567

[B81] Zhang M. Zhang F. Liu T. Shao P. Duan L. Yan J. (2020). <p&gt;Polydopamine nanoparticles camouflaged by stem cell membranes for synergistic chemo-photothermal therapy of malignant bone tumors</p&gt;. Int. J. nanomedicine 15, 10183–10197. 10.2147/ijn.s282931 33363374 PMC7754090

[B82] Zhang W. Wang Z. Huang J. Jiang Y. (2021). Zirconia-based solid acid catalysts for biomass conversion. Energy & Fuels 35, 9209–9227. 10.1021/acs.energyfuels.1c00709

[B83] Zhang Y. Wang X. (2020). Targeting the Wnt/β-catenin signaling pathway in cancer. J. Hematol. Oncol. 13, 165. 10.1186/s13045-020-00990-3 33276800 PMC7716495

[B84] Zhang Z. Wang J. Chen C. (2013). Near-infrared light-mediated nanoplatforms for cancer thermo-chemotherapy and optical imaging. Adv. Mater. 25, 3869–3880. 10.1002/adma.201301890 24048973

[B85] Zhao J. Mu X. Hou X. Zhang X. Li P. Jiang J. (2023b). Synergistic treatment of osteosarcoma with biomimetic nanoparticles transporting doxorubicin and siRNA. Front. Oncol. 13, 1111855. 10.3389/fonc.2023.1111855 36756155 PMC9900173

[B86] Zhao T.-T. Zhou T.-J. Zhang C. Liu Y.-X. Wang W.-J. Li C. (2023a). Hypoxia inhibitor combined with chemotherapeutic agents for antitumor and antimetastatic efficacy against osteosarcoma. Mol. Pharm. 20, 2612–2623. 10.1021/acs.molpharmaceut.3c00068 37042832

[B87] Zheng G. Z. Zhang Q. H. Chang B. Xie P. Liao H. Du S. X. (2023). Dioscin induces osteosarcoma cell apoptosis by upregulating ROS-mediated P38 MAPK signaling. Drug Dev. Res. 84, 25–35. 10.1002/ddr.22009 36401839

[B88] Zhong J. Wen W. Wang J. Zhang M. Jia Y. Ma X. (2023). Bone-Targeted dual functional lipid-coated drug delivery system for osteosarcoma therapy. Pharm. Res. 40, 231–243. 10.1007/s11095-022-03430-8 36380167 PMC9666974

[B89] Zhou X. Moussa F. M. Mankoci S. Ustriyana P. Zhang N. Abdelmagid S. (2016). Orthosilicic acid, Si(OH)4, stimulates osteoblast differentiation *in vitro* by upregulating miR-146a to antagonize NF-κB activation. Acta Biomater. 39, 192–202. 10.1016/j.actbio.2016.05.007 27163405

[B90] Zhu P. Li T. Li Q. Gu Y. Shu Y. Hu K. (2022). Mechanism and role of endoplasmic reticulum stress in osteosarcoma. Biomolecules 12, 1882. 10.3390/biom12121882 36551309 PMC9775044

[B91] Zong Q. Li J. Xiao X. Du X. Yuan Y. (2022). Self-amplified chain-shattering cinnamaldehyde-based poly(thioacetal) boosts cancer chemo-immunotherapy. Acta Biomater. 154, 97–107. 10.1016/j.actbio.2022.09.066 36210042

